# Supplementary material for: Resolving the full spectrum of human genome variation using Linked-Reads
Source: Genome Res. 2019 Apr;29(4):635–45. doi: 10.1101/gr.234443.118 (PMC6442396; doi:10.1101/gr.234443.118)
Supplement: Supplemental Material [file supp_gr.234443.118_Supplemental_longranger-2.2.zip › longranger-2.2.tar.bz2!/lr2.2-public/lib/stan/cmdstan/stan/doc/api/html/classstan_1_1optimization_1_1_convergence_options.html]

Stan: stan::optimization::ConvergenceOptions< Scalar > Class Template Reference


|  |  |
| --- | --- |
|  | Stan  2.5.0  probability, sampling & optimization |


- Main Page
- Related Pages
- Namespaces
- Classes
- Files

- Class List
- Class Index
- Class Hierarchy
- Class Members

All Classes Namespaces Files Functions Variables Typedefs Enumerations Enumerator Friends Macros Pages

- stan
- optimization
- ConvergenceOptions

Public Member Functions |
Public Attributes |
List of all members

stan::optimization::ConvergenceOptions< Scalar > Class Template Reference

`#include <bfgs.hpp>`

|  |  |
| --- | --- |
| Public Member Functions | |
|  | ConvergenceOptions () |
|  | |

|  |  |
| --- | --- |
| Public Attributes | |
| size\_t | maxIts |
|  | |
| Scalar | tolAbsX |
|  | |
| Scalar | tolAbsF |
|  | |
| Scalar | tolRelF |
|  | |
| Scalar | fScale |
|  | |
| Scalar | tolAbsGrad |
|  | |
| Scalar | tolRelGrad |
|  | |

## Detailed Description

### template<typename Scalar = double> class stan::optimization::ConvergenceOptions< Scalar >

Definition at line 33 of file bfgs.hpp.

## Constructor & Destructor Documentation

template<typename Scalar = double>

|  |  |  |  |  |
| --- | --- | --- | --- | --- |
| stan::optimization::ConvergenceOptions< Scalar >::ConvergenceOptions | ( |  | ) |  |

inline

Definition at line 35 of file bfgs.hpp.

## Member Data Documentation

template<typename Scalar = double>

Scalar stan::optimization::ConvergenceOptions< Scalar >::fScale

Definition at line 50 of file bfgs.hpp.

template<typename Scalar = double>

size\_t stan::optimization::ConvergenceOptions< Scalar >::maxIts

Definition at line 46 of file bfgs.hpp.

template<typename Scalar = double>

Scalar stan::optimization::ConvergenceOptions< Scalar >::tolAbsF

Definition at line 48 of file bfgs.hpp.

template<typename Scalar = double>

Scalar stan::optimization::ConvergenceOptions< Scalar >::tolAbsGrad

Definition at line 51 of file bfgs.hpp.

template<typename Scalar = double>

Scalar stan::optimization::ConvergenceOptions< Scalar >::tolAbsX

Definition at line 47 of file bfgs.hpp.

template<typename Scalar = double>

Scalar stan::optimization::ConvergenceOptions< Scalar >::tolRelF

Definition at line 49 of file bfgs.hpp.

template<typename Scalar = double>

Scalar stan::optimization::ConvergenceOptions< Scalar >::tolRelGrad

Definition at line 52 of file bfgs.hpp.

---

The documentation for this class was generated from the following file:

- src/stan/optimization/bfgs.hpp


---

[ Stan Home Page ]

*© 2011–2014,
Stan Development Team.*
